# Medical and Philosophical Intelligence

**Published:** 1818-09

**Authors:** 


### MEDICAL AND PHILOSOPHICAL INTELLIGENCE.

*On Meteoric Stones.* From Mr. BRANDE's Lectures at the Royal Institution.

THE first tolerably accurate narration of the fall of a meteoric stone relates to that of Ensisheim, near Basle, upon the Rhine. The account, which is deposited in the church, was this:—A. D. 1492, Wednesday, 7th November, there was a loud clap of thunder, and a child saw a stone fall from heaven; it struck into a field of wheat, and did no harm, but made a hole there. The noise it made was heard at Lucerne, Villing, and other places. On the Monday, King Maximilian ordered the stone to be brought to the castle; and, after having conversed about it with the noblemen, said the people of Ensisheim should hang it up in their church, and his Royal Excellency strictly forbade any body to take any thing from it. *His Excellency, however, took two pieces himself, and sent another to Duke Sigismund of Austria.* This stone weighed 255 lbs.

In 1627, 27th November, the celebrated Gassendi saw a burning stone fall on Mount Vaisir, in Provence; he found it to weigh 59 lbs.

In 1672, a stone fell near Verona, weighing 300 lbs. And Lucas, when at Larissa, 1706, describes the falling of a stone, with a loud hissing noise, and smelling of sulphur.

In September, 1753, De Lalande witnessed this extraordinary phenomenon, near Pont de Vesli. In 1768, no less than three stones fell in different parts of France. In 1790, there was a shower of stones near Agen, witnessed by M. Darcet, and several other respectable persons. And, on the 18th of December, 1795, a stone fell near Major Topham's house in Yorkshire: it was seen by a ploughman and two other persons, who immediately dug it out of the hole it had buried itself in; it weighed 56 lbs.

We have various other, and equally satisfactory, accounts of the same kind. All concur in describing a luminous meteor moving through the air in a more or less oblique direction, attended by a hissing noise, and the fall of stony or semi-metallic masses, in a state of ignition. We have, however, evidence of another kind, amply proving the peculiarities of these bodies. It is, that, although they have fallen in very different countries, and at distant periods, when submitted to chemical analysis, they all agree in component parts: the metallic particles being composed of nickel and iron; the earthy, of silex and magnesia.

Large masses of native iron have been found in different parts of the world, of the history and origin of which nothing very accurate is known. Such are the great block of iron at Elbogen, in Bohemia; the large mass discovered by Pallas, weighing 1600 lbs. near Krasnojark, in Siberia; that found by Goldberry, in the great desert of Zahra, in Africa; probably also that mentioned by Mr. Barrow, on the banks of the great fish-river in Southern Africa; and those noticed by Bruce, Bougainville, Humboldt, and others, in America, of enormous magnitude, exceeding thirty tons in weight. That these should be of the same source as the other meteoric stones seems at first to startle belief; but, when they are submitted to analysis, and the iron they contain found alloyed by nickel, it no longer seems credulous to regard them as of meteoric origin. We find nothing of the kind in the earth.

To account for these uncommon visitations of metallic and lapideous bodies, a variety of hypotheses have been suggested.

Are they merely earthly matter fused by lightning? Are they the offspring of any terrestrial volcano? These were once favourite notions; but we know of no instance in which similar bodies have in that way been produced; nor do the lavas of known volcanoes in the least resemble these bodies, to say nothing of the inexplicable projectile force that would here be wanted. This is merely explaining what is puzzling, by assuming what is impossible; and the persons who have taken up this conjecture have assumed one impossibility to account for what they conceive to be another,—namely, that the stony bodies should come from any other source than our own globe.

The notion that these bodies come from the moon, though it has been laughed at as lunacy, is, when impartially considered, neither absurd nor impossible. It is quite true that the quiet way in which they visit us is against such an origin: it seems, however, that any power which would move a body 6000 feet in a second, (that is, about three times the velocity of a cannon-ball,) would throw it from the sphere of the moon's attraction into that of our earth. The cause of this projective force may be a volcano; and, if thus impelled, the body would reach us in about two days, and enter our atmosphere with a velocity of about 25,000 feet in a second. Their ignition may be accounted for, either by supposing the heat generated by their motion in our atmosphere sufficient to ignite them; or by considering them as combustibles, ignited by the mere contact of air.

While we are considering the possibility of these considerations, it may be remembered that, in the great laboratory of the atmosphere, chemical changes may happen, attended by the production of iron and other metals; that, at all events, such a circumstance is within the range of possible occurrences; and that the meteoric bodies, which thus salute the earth with stony showers, may be children of the air, created by the union of simpler forms of matter. The singular relationship between iron, and nickel, and magnetism, and the uniform influence of meteoric phenomena upon the magnetic needle, should be taken into the account in these hypotheses.

---

At the Quarterly Court held last month, the following were the officers of the *Royal College of Surgeons* chosen for the ensuing year:—

*Master*.—*Thomas Keate, Surgeon to the Queen and Prince Regent.

*Governors*.—*Sir David Dundas, Bart. Serjeant-surgeon to the King; *Thompson Forster.

*George Chandler; John Heaviside, Surgeon-Extraordinary to the King; *Sir William Blizard, Knt.; *Henry Cline; *William Norris; *Sir Everard Home, Bart. Sergeant-Surgeon to the King; John Adair Hawkins; Francis Knight; *Sir Ludford Harvey, Knt.; *William Lynn; John Abernethy; William Lucas; Astley P. Cooper; Anthony Carlisle, Surgeon-Extraordinary to the Prince Regent; Thomas Chevalier, Surgeon-Extra to the Prince Regent; James Wilson; John Gunning, Surgeon-Extraordinary to the King; H. L. Thomas.

*Those marked thus * are Examiners.*

---

The following Gentlemen have obtained the degree of Doctor in Medicine from the university of Glasgow within the last twelve months:—Mr. John Gemmell, from Scotland; Mr. John Campbell, from ditto; Mr. John Easton, from ditto; Mr. James

Paterson, from ditto; Mr. Wm. Barry, from England; Mr. Thos. Cartan, from Ireland; Mr. Wm. Jones, from England; Mr. Jenkin Jones, from ditto; Mr. Edw. Willoughby, from Barbadoes; Mr. Edward O'Reilly, from Ireland; Mr. James Steel, from Scotland; Mr. Wm. Drew, from ditto.

---

*Account of the Complete School of Physic in Ireland, for the Instruction of Students in Medicine, Surgery, and Pharmacy.*

This hospital is chiefly supported by the rents of Sir P. Dunn's estates, and partly by private contribution. The Board of Governors consist of the visitors of the College of Physicians, the president, vice-president, and censors of the same, the provost of Trinity College, and twelve subscribers; but no physician or surgeon of the hospital is eligible to be a governor. The house is intended to hold 130 patients; of whom thirty are selected for instruction and lectures, by the Clinical professor for the time; the rest are placed under the care of a physician appointed by the governors.

The several students in physic are matriculated in the University, for which they pay five shillings; but such students, unless they shall think proper, are not obliged to attend to the academical duties of the University. The several lecturers, when they have delivered one half of their courses, return to the senior lecturer of Trinity College a list of such pupils as shall have attended them during such part of their courses.

There are six professorships. Those of Anatomy and Surgery, of Chemistry, and of Botany, are on the foundation of Trinity College, and are called the University professorships; those of the Institutes of Medicine, of the Practice of Medicine, and of Materia Medica and Pharmacy, are on Sir Patrick Dunn's foundation, and are named King's professorships.

A large collection of medical books, bequeathed by Sir P. Dunn, is appropriated to the use of the students; and provision is made for purchasing books in proportion as the funds increase.

The students who do not graduate in Arts are permitted, at the end of three years from the date of their matriculation, to undergo an examination before the six professors of the school, in their respective departments, on producing to the Board of Trinity College certificates of diligent and regular attendance on Anatomy, Surgery, Chemistry, Botany, Institutes of Medicine, Practice of Medicine, Materia Medica and Pharmacy, the Clinical Lectures, and practice of Sir Patrick's Dunn's Hospital. They likewise write a Thesis in Latin.

The students who go through a collegiate course, on producing certificates of their strict attendance on the lectures of the professors in the School of Physic, on the Clinical Lectures, and the Hospital, are, three years after having graduated as Bachelor of Arts, admitted to an examination before the Regius Professor of Physic, and the Professors of Anatomy and Surgery, Chemistry, and Botany, in Trinity College. On being approved, and performing the usual academical exercises, they take the degree of Bachelor of Medicine. Upon sufficient standing, publishing a Thesis, passing a second examination before the University professors, and performing the necessary acts, the full degree of Doctor in Medicine is conferred. These rank with the degrees of Bachelor and Doctor of Medicine obtained in the Universities of Oxford and Cambridge.

---

*Brief Account of the Imperial Society of Naturalists, at Moscow.* By Dr. Lyall, Physician to the Countess Orlof-Tchesminsky.

The plan for forming a depôt for the discoveries in natural history, in the vast empire of Russia, and uniting the friends of this science who wished to lend their assistance for that purpose, and of publishing in Russia the history of the discoveries made in the empire, was conceived by Professor Gotthelf Fischer, on his arrival at Petersburgh, in the year 1804. It was not till the summer of 1805, however, that a few of the professors of the University of Moscow and of the literati first assembled, and adopted the regulations proposed by Professor Fischer.

The object of the Society is to occupy itself with natural history and the relative sciences,—as human and comparative anatomy, chemistry, natural philosophy, rural economy, &c. &c.

The Society consists of members *ordinary* and *honorary*. The ordinary members are divided into *resident* and *non-resident*. Shortly after the association above-mentioned took place, his Excellency the late Mr. Mouraviof, curator of the University, and colleague of the Minister of Public Instruction, being informed that the Society had begun to meet at the house of the director, Professor Fischer, presented its regulations to his Imperial Majesty the Emperor Alexander, who approved of the plan, and therefore ordered Mr. Mouraviof to testify his gracious satisfaction and approbation to the Professor.

Soon after the institution of the Society, the literati, and particularly the cultivators of natural history, including many of the nobility in Petersburgh and Moscow, and in the other towns and universities in Russia, many of the most distinguished philosophers and naturalists on the continent, chiefly through the extensive acquaintance of the founder and director, were enrolled among its members. Presents were received from all quarters, of books, objects of natural history, and money. The Society was very flourishing; and, by the year 1812, had published four volumes of its Transactions in different languages. All the collections of the Society were deposited in the museum of the University, and, along with that extensive collection, became a common prey to the flames in the memorable year 1812. Among other things were lost some manuscripts, and almost the whole of the impression of the four volumes of the Transactions, and also the second edition of the first volume. Far from being dispirited by this irreparable misfortune, (particularly to Professor Fischer, who had arranged and published a Catalogue, in three volumes, of the Museum Demidof, and two volumes of the Imperial Museum, now united,) he and the other members re-assembled in 1813, began their proceedings, and since that period have continued all their efforts with unremitting vigour to recover from their losses. The Society has again collected a small museum, which has been considerably augmented this winter; as well as the library, consisting of above 300 volumes. (The library of the University is restored to the amount of 7000 volumes.)

The Society unites here a number of men of great, and others of considerable, talents, whose works are too little known in Great Britain. From the change in the state of Europe, however, a more free interchange of scientific publications is to be wished for, and may be expected. The Society intends very soon to reprint the four volumes of its Transactions; and in the mean time the fifth volume is in the press, which forms the first of a new collection. The whole of these volumes are replete with matter important to the natural historian and philosopher.

The number of the Society's honorary and non-resident ordinary members is very great, and includes the most distinguished characters in Europe and America, &c.

The Imperial Society of Naturalists is well known on the continent, and is desirous of becoming better known also in Great Britain by an interchange of its Transactions for the Transactions of the Natural History and Literary Societies of our island, as well as to receive the donations of objects of natural history, or of books, from its members, or from individuals disposed to assist its views.

Societies wishing to make an exchange of Transactions may address themselves as follows:—"To Professor Gotthelf Fischer, at Moscow." Or, if they will send a copy of their Transactions, they may rely with perfect confidence that Professor Fischer will return in their place the works of the Imperial Society of Naturalists.

---

A Memorial, by Messrs. Pelletier, professor of Pharmacy, and Caventon, apothecary to St. Anthony's Hospital, was read at the Royal Institute at Paris, the 20th April, 1818; exhibiting the result of many experiments, made with the intention of discovering, not only the chemical composition of cochineal, but also to separate, perfectly, the colouring matter of this precious substance. Messrs. Pelletier, and his worthy pupil Caventon, have completely succeeded in this inquiry; the result of which shews that cochineal is composed—

1. Of carmine, the name they give to the colouring matter;
2. Of a peculiar animal substance;
3. Of an oily matter, composed of stéarine, eixine, and an odoriferous acid;
4. Of phosphate and carbonate of lime and potash, combined with a peculiar acid.

*On the Colours of Bodies ;* by M. Prevost.

A new hypothesis respecting the cause of colour in bodies has been lately proposed by M. Ben. Prevost, according to which it is supposed that the effect depends not upon reflection, but upon radiation. It was formerly supposed that the different rays which compose white light, were all of them, except those which produce the colour of the body, absorbed by it, whilst these were reflected; M. Prevost, however, conceives that coloured bodies reflect a portion of the light in its white or compound state, and that they decompose a part of that which penetrates their substance into two new parts; one of which remains in the body, and the other radiates from all parts of their surface. The colour of bodies, as we commonly see it, is rendered pale by the white light which is mixed with it; but it may be deprived of this by a series of mutual reflections and decompositions, so as considerably to augment the intensity of the colour. If, for example, we receive successively the image of a plate of gold, which is polished and illuminated by a bright light, upon a second plate, and this image upon a third, &c. we may, after twelve or eighteen of these successive reflections, procure a deep-red orange, which is probably the real colour of gold. By applying the same process to copper, we procure a colour which approaches to that of scarlet; silver becomes of a beautiful yellow; tinned iron exhibits a yellow deeper than that which is generally ascribed to gold: and, in short, M. Prevost concludes from his experiments that there is no metal which is properly white or grey, but that they all of them possess some decided brilliant colour.

---

*On the new Metal Cadmium ;* by M. Gay-Lussac.

The new metal resembles tin in its colour, its lustre, its softness, its ductility, and the sound which it produces when it is bent. It melts and volatilizes at a temperature a little lower than zinc. It preserves its splendour in the air ; but by heat it is changed into an orange yellow oxide, which is not volatile, and which is very easily reduced. This oxide does not colour borax; it dissolves very readily in acids, and forms colourless salts, from which it is precipitated white by alkalies. The hydrosulphuric acid precipitates it yellow, like arsenic. Zinc precipitates it in the metallic state. It specific gravity at 77° (F.) is 8·635.

The metal was discovered in the autumn of the last year, by M. Stromeyer, while he was officially examining the apothecaries' shops at Hanover. M. Hermann, who prepares this oxide on the great scale for medicinal purposes, having been prohibited from selling it, (because the presence of arsenic had been supposed to have been detected in it) particularly examined it, and perceived that it contained a new body, which he procured in a separate state, and sent to M. Stromeyer, begging him to verify his conjectures. M. Stromeyer soon found that it had the same properties with the metal which he had just discovered, to which he gives the name of Cadmium.—*Ann de Chimie et de Phys.*

### *Influence of Music on the Mouse.*

Dr. Archer, of Norfolk, America, has published an account of a circumstance, which tends to confirm the truth of the observation of Linnæus on this subject; he says—

"On a rainy evening, in the winter of 1815, as I was alone in my chamber, I took up my flute, and commenced playing. In a few minutes my attention was directed to a mouse that I saw creeping from a hole, and advancing towards the chair I was sitting in. I ceased playing, and it ran precipitately back to its hole. I began again shortly afterwards, and was much surprised to see it reappear, and take its old position. The appearance of the little animal was truly delightful, and at the same time wonderful. It couched itself on the floor, shut its eyes, and appeared to be in ecstasy. I ceased playing, and it instantly disappeared again.

"This experiment I repeated frequently, with the same success; observing that it was always differently affected, as the music varied from the slow and plaintive to the brisk or lively. It finally went off, and all my art could not entice it to return.

"This case may add some weight to the evidence of others on this subject, particularly as it is held in derision by many; and Gmelin himself, not believing, with Linnæus, that 'mus delectatur musica,' has omitted this part of its history from his edition of that author's Systema Naturæ."—*Amer. Med. Recorder*, vol. i. No. 1.

A more remarkable instance of this fact was inserted in the *Philadelphia Medical and Physical Journal*, p. 37, communicated by Dr. Cramer, of Jefferson County. The circumstance, he says, was related to him by a gentleman of undoubted veracity.

"One evening, in the month of December, as a few officers on board of a British man-of-war in the harbour of Portsmouth were seated round the fire, one of them began to play a plaintive air on the violin. He had scarcely performed ten minutes, when a mouse, apparently frantic, made its appearance in the centre of the floor, near the large table which usually stands in the ward-room, the residence of the lieutenants in ships of the line. The strange gestures of the little animal strongly excited the attention of the officers, who, with one consent, resolved to suffer it to continue its singular actions unmolested. Its exertions now appeared to be greater every moment: it shook its head, leaped about the table, and exhibited signs of the most ecstatic delight.

"It was observed that, in proportion to the gradation of the tones to the soft point, the ecstacy of the animal appeared to be increased, and *vice versa*. After performing actions which an animal so diminished would, at first sight, seem incapable of, the little creature, to the astonishment of the delighted spectators, suddenly ceased to move, fell down, and expired, without evincing any symptoms of pain."

Facts somewhat similar to the preceding, but not (that I know) so circumstantial, are recorded by different authors. Linnæus notices the circumstance in two words. Speaking of the common mouse (mus musculus), he says, "delectatur musica."—*Systema Naturæ*, &c. tom. i. p. 83, No. 13. Gmelin, in his edition of the System, omits this part of the Linnæan history of the animal.

### *On the Milk of the Cow-Tree, and on Vegetable Milk in general.* By M. Humboldt.*

M. Humboldt and his companions, in the course of their travels, heard an account of a tree which grows in the valleys of Aragua, the juice of which is a nourishing milk, and which, from that circumstance, has received the name of the cow-tree. As the milky juices of plants are in general acrid, bitter, and even poisonous, M. Humboldt was at first scarcely disposed to credit the account; but experience proved it to be correct.

The tree, in its general aspect, resembles the chrysophyllum cainito: its leaves are oblong, pointed, leathery, and alternate, marked with lateral veins, projecting downwards; they are parallel, and are ten inches long. M. Humboldt had no opportunity of seeing the flower; the fruit is somewhat fleshy, and contains one, or sometimes two, nuts. When incisions are made into the trunk, it discharges abundantly a glutinous milk, moderately thick, without any acridness, and exhaling an agreeable balsamic odour. The travellers drank considerable quantities of it without experiencing any injurious effects; its viscidity only rendering it rather unpleasant. The superintendent of the plantation assured them that the negroes acquire flesh during the season in which the cow-tree yields the greatest quantity of milk.

When this fluid is exposed to the air, perhaps in consequence of the absorption of the oxygen of the atmosphere, its surface becomes covered with membranes of a substance that appears to be of a decided animal nature, yellowish, thready, and of a cheesy consistence. These membranes, when separated from the more aqueous part of the fluid, are almost as elastic as caoutchouc; but, at the same time, they are as much disposed to become putrid as gelatine. The natives give the name of cheese to the coagulum, which is separated by the contact of the air; in the course of five or six days it becomes sour. The milk, kept for some time in a corked phial, had deposited a little coagulum, and still exhaled its balsamic odour. If the recent juice be mixed with cold water, the coagulum is formed in small quantity only; but the separation of the viscid membranes occurs when it is placed in contact with nitric acid.

This remarkable tree seems to be peculiar to the Cordilliere du Littoral, especially from Barbula to the lake of Maracaybo. There are likewise some traces of it near the village of San Mateo; and, according to the account of M. Bredmeyer, in the village of Caucagua, three days' journey to the east of the Caraccas. This naturalist has likewise described the vegetable milk of the cow-tree as possessing an agreeable flavour and an aromatic odour: the natives of Caucagua call it the milk-tree.

---

* Abridged from an Essay in the Annals de Chimie, for February, 1818, which is an extract from a memoir read to the Academy of Sciences.

*Observations on Tetanus, Medical and Surgical.* Communicated to the *Société Medicale d'Emulation*, at Paris, by Dr. Louis Franck, Privy-Counsellor and first Physician to her Highness the Duchess of Parma.

The disappointment experienced by anatomists in their inquiries into the origin of this disease, after having been long expressed in useless regret, has at length excited a degree of ardour for the investigation that promises to lead to the most important results. Dr. John Franck first called the attention of physicians to the spinal marrow in a memorial on this subject, published in 1791; and his views have been adopted, and the truth of his observations generally acknowledged, throughout Europe. Professor Rachette, a short time since, published an interesting Treatise on Diseases of the Spinal Marrow; and Dr. Copeland produced, in 1815, a very important work on the same subject. Dr. Franck relates two cases that have lately occurred to the observation of Professor Brera of Padua. The first occurred in a woman, aged twenty-two; it appeared to arise without any evident cause: all the usual remedies were tried without success; she died on the fifteenth day. On dissection, a considerable quantity of pus was found in the cavity of the abdomen, the consequence of general peritoneal inflammation; the spinal marrow was soft and considerably altered in structure; there was effusion of serum between its coats, and the vessels were much distended with blood.

The subject of the second case was a young man, aged nineteen, who had received a contusion on the thumb of the right hand: twelve days after, he began to feel a stiffness in the motions of the lower jaw, which gradually increased; and, in a few days, he was seized with tetanus. When brought to the hospital, he was covered with a cold sweat, and complained of pain over the whole body, particularly about the lumbar region and along the spinal canal; the face was red and flushed, and the pulse small and contracted. Professor Brera directed a hundred and twenty leeches to be applied along the course of the spine, and prussic acid to be exhibited internally. After the leeches had been applied, relaxation of the muscles came on; but this was succeeded by paralysis, and he soon afterwards expired.

The examination of the body after death was highly interesting: there had been violent inflammation of the spinal marrow, commencing at the situation of the lower cervical vertebræ, and continued to the extremity. But, what was very remarkable, the inflammation was confined to the right side of the spinal marrow, corresponding in this respect with the hand injured.

### REPORT OF DISEASES.

The variation in the temperature of the atmosphere, which has taken place during the last month, has been productive of a greater diversity of character in the prevalent diseases than is usual at this season.

At the early part of the month, suffusion of blood in the head, and bilious diarrhœa, were the most common complaints. We witnessed a few cases of continued fever, which soon put on the character of typhus mitior, that were unaccompanied by any evident signs of local inflammation: these did well under the use of the cold affusion, and the free exhibition of cold drink.

Some severe forms of fever have occurred since the fourteenth, when the wind began to blow from the north with an unusual degree of coldness; inflammation of the brain, or some of the abdominal viscera, has usually accompanied the onset of fever, which has required free and repeated blood-letting for its removal: we have been astonished at the celerity with which inflammation has run its course to a fatal termination in those cases in which the most vigorous measures for its relief were not had recourse to in the first instance. It appears that the long and excessive heat has, in many instances, been productive of the state of body common in hot climates, which is so ill adapted to tolerate fever and inflammatory action. One young man, aged 25, was seized with rigor, followed with burning skin, oppressive head-ache, and the most complete general langour. After losing twenty ounces of blood, and being well purged, &c. he felt perfectly relieved; but, the following day, the same symptoms, without the head-ache, were accompanied with a pain in the course of the spinal marrow, which became exquisite on attempting to turn in bed: free bleeding and the usual antiphlogistic remedies were employed, by which he was again relieved; the pulse became tolerably soft, and the skin moist. After passing a good night, he was attacked with inflammation of the bowels, which was removed by another general bleeding, a dozen leeches, and the production of a blister on the abdomen. For three or four days from this time he appeared to be going on well; he had a slight degree of fever, but slept well, and was regaining his appetite, when he was seized with pain and tension of the bowels: this continued for about *two hours*, and was finally removed on going into a warm bath. He, however, after this, began to sink, and expired the following day. On dissection, about two pints of pus were found in the cavity of the abdomen, proceeding from general inflammation of the peritoneum. There was not the least appearance of gangrene in any part of the intestines; indeed, they had performed their functions until a few hours b fore death. The brain and spinal marrow were in a healthy state.

What is usually called typhus mitior has appeared in some of the schools in the neighbourhood of London, and has proved fatal in some cases where the symptoms of local inflammation have been neglected or improperly treated.

Measles has also become prevalent; but the disease is, for the most part, mild, and unattended by any serious affection of the lungs.

### METEOROLOGICAL JOURNAL,

**By Messrs. William Harris and Co. 50, Holborn, London.**

***From the 20th July to the 19th August, inclusive.***

| Day of Month. | Moon. | Rain gauge | Therm. | | | Barom. | | De Luc's Hygrom. Dry | | Damp | | Wind. | | Atmospheric Variation. | | |
|---|---|---|---|---|---|---|---|---|---|---|---|---|---|---|---|---|
| July | | | | | | | | | | | | | | | | |
| 20 | | | 75 | 80 | 65 | 29·99 | 29·93 | | | 10 | 7 | E | E | Fine | | |
| 21 | | | 58 | 75 | 61 | 29·90 | 29·95 | | | 11 | 13 | E | NE | Fine | Clo. | |
| 22 | | | 67 | 75 | 62 | 30·00 | 30·08 | | | 10 | 10 | W | SW | Fine | | |
| 23 | | | 69 | 81 | 68 | 29·97 | 29·95 | | | 3 | 5 | WNW | NE | Fine | | |
| 24 | | | 75 | 84 | 68 | 29·82 | 29·77 | | | 3 | 5 | SSE | S | Fine | | Light. & Rain |
| 25 | ☾ | ,15 | 73 | 80 | 67 | 29·90 | 29·75 | | | 7 | 12 | SE | S | Fine | | Clo. |
| 26 | | | 70 | 80 | 63 | 29·95 | 29·93 | | | 10 | 12 | S | SW | Fine | | Rain |
| 27 | | ,07 | 68 | 81 | 60 | 29·81 | 30·05 | | | 15 | 13 | SW | NW | Fine | Rain | Clo. |
| 28 | | ,04 | 63 | 78 | 60 | 29·91 | 30·22 | | | 5 | 3 | SW | SW | Fine | | |
| 29 | | | 65 | 76 | 62 | 30·20 | 30·16 | | | 7 | 7 | SW | SW | Clo. | Fine | Clo. |
| 30 | | | 67 | 74 | 63 | 30·10 | 30·07 | | | 7 | 5 | W | SW | Clo. | Fine | |
| 31 | | ,03 | 67 | 75 | 61 | 29·96 | 29·97 | | | 8 | 10 | SSW | SW | Clo. | Fine | Showery |
| Aug. | | | | | | | | | | | | | | | | |
| 1 | | | 65 | 70 | 58 | 30·00 | 30·07 | | | 12 | 15 | NW | NW | Clo. | Fine | |
| 2 | ● | | 60 | 70 | 55 | 30·03 | 30·11 | | | 10 | 10 | NNE | SE | Fine | | |
| 3 | | | 58 | 68 | 55 | 30·08 | 30·06 | | | 9 | 7 | SE | SE | Fine | | |
| 4 | | | 60 | 75 | 60 | 30·08 | 30·07 | | 0 | 5 | 0 | ESE | SE | Fog | Fine | |
| 5 | | | 67 | 80 | 69 | 30·06 | 30·01 | 0 | 3 | 0 | | S | SSW | Fine | | |
| 6 | | | 76 | 84 | 60 | 30·03 | 30·06 | 0 | | 0 | 5 | NE | NNE | Fine | | Lightning |
| 7 | | | 69 | 79 | 62 | 30·06 | 30·05 | | | 8 | 9 | NW | W | Fine | | |
| 8 | | | 69 | 78 | 60 | 30·00 | 30·02 | | | 10 | 9 | SSW | SW | Clo. | Fine | |
| 9 | ☽ | ,02 | 68 | 75 | 59 | 29·95 | 29·90 | | | 9 | 11 | SW | ENE | Rain | Fine | |
| 10 | | | 64 | 75 | 56 | 29·97 | 30·00 | | | 5 | 7 | ENE | ENE | Fine | | |
| 11 | | | 64 | 68 | 56 | 30·14 | 30·10 | | | 9 | 5 | NNE | NE | Fine | | Clo. |
| 12 | | | 60 | 69 | 53 | 30·13 | 30·10 | | | 5 | 7 | NE | NE | Fine | | |
| 13 | | | 58 | 69 | 50 | 30·10 | 29·99 | | | 7 | 7 | NE | NE | Fine | | |
| 14 | | | 57 | 69 | 56 | 29 97 | 30·00 | | | 10 | 9 | NE | NE | Fine | | |
| 15 | | | 59 | 72 | 60 | 30·05 | 30·09 | | | 10 | 7 | NE | NE | Fine | | |
| 16 | ○ | | 67 | 74 | 60 | 30·08 | 30·03 | | | 7 | 5 | NW | NE | Fine | | |
| 17 | | | 63 | 71 | 59 | 30·00 | 29 99 | | | 3 | 7 | NW | NW | Fine | | |
| 18 | | | 63 | 70 | 60 | 30·00 | 30·00 | | | 7 | 10 | N | N | Fine | | |
| 19 | | | 61 | 65 | 57 | 30 00 | 30 00 | | | 10 | 9 | N | N | Fine | | |

The quantity of rain fallen in the month of July is 59-100ths.

