# Errata

**Published:** 1818-12

**Authors:** 


					259, ? 1, dele Medical and ourgtcul.

				

